# Cost-utility analysis of repetitive transcranial magnetic stimulation as add-on therapy to standard care for the treatment of hallucinations in schizophrenia

**DOI:** 10.1192/j.eurpsy.2022.13

**Published:** 2022-03-30

**Authors:** Lauren Hendriks, Cathrine Mihalopoulos, Long Khanh-Dao Le, Colleen Loo, Mary Lou Chatterton

**Affiliations:** 1 Deakin Health Economics, Institute for Health Transformation, Deakin University, Geelong, Victoria, Australia; 2 School of Psychiatry, University of New South Wales, Sydney, New South Wales, Australia

**Keywords:** Cost-utility analysis, disability-adjusted life-years, repetitive transcranial magnetic stimulation, schizophrenia

## Abstract

**Background:**

This research evaluates the cost-effectiveness of repetitive transcranial magnetic stimulation (rTMS) as add-on therapy to standard care for adults with schizophrenia from an Australian health system perspective.

**Methods:**

A Markov model estimated costs in 2021 Australian dollars and Disability-Adjusted Life-Years (DALYs) averted with rTMS added to standard care compared to standard care alone over 12-months for adults aged 25–65 years with hallucinations in schizophrenia refractory to other therapies. rTMS effect size was sourced from a meta-analysis and converted to a relative risk using the Cochrane conversion method. Probabilistic sensitivity analysis evaluated uncertainty in effect size and disability weights. One-way sensitivity analyses varied rTMS session cost and effectiveness, time horizon and inpatient costs.

**Results:**

The base-case average incremental cost-effectiveness ratio (ICER) was $87,310/DALY averted (95% UI: $10,157–$97,877). Reducing rTMS session cost to $100 lowered the ICER to $9,127/DALY (95% UI: Dominant–$50,699). A 4-year time horizon resulted in rTMS being less costly and more effective (Dominant) than standard care. Decreasing the 3-month probability of relapse with rTMS to 4.6% resulted in a 71% probability of rTMS being cost-effective.

**Conclusions:**

Using a threshold of $50,000/ DALY averted, rTMS as add-on therapy to standard care for the treatment of refractory hallucinations in schizophrenia would not be considered a cost-effective treatment option compared to standard care alone. However, given the refractory nature of this condition and the relatively small size of this population, it may be reasonable for decision-makers to adopt a higher ICER threshold.

## Introduction

The global prevalence of schizophrenia has been estimated at 0.28% and ranked globally as the 12th most disabling disorder [1]. In Australia, the economic impact of psychosis from a societal perspective was estimated at over $77,000 per person (2010 Australian dollars [AUD]), for a total of $4.9 billion per year [2].

For people with a diagnosis of schizophrenia who have persistent, established illness or unremitted disease, ongoing treatment aims to prevent and manage acute relapse. Standard care includes trials of one or more antipsychotic medications, including clozapine or long-acting injectable antipsychotics, in addition to cognitive behavioral therapy (CBT) [3]. Despite optimized pharmacological treatment, a proportion of patients continue to experience disease symptoms [4]. Australian guidelines note repetitive transcranial magnetic stimulation (rTMS) is considered an effective and safe treatment for auditory hallucinations and negative symptoms while transcranial direct current stimulation (tDCS) has limited evidence of effectiveness [3, 5].

Despite the evidence supporting the use of rTMS for people with schizophrenia, it is not routinely administered in the Australian treatment setting. rTMS treatment has a favorable safety profile, does not need to be limited to administration in the hospital inpatient setting, and if public subsidy were available, would allow the service to be offered more broadly, improving access and equity.

Therefore, the aim of the current study was to evaluate the cost-effectiveness of rTMS as an addition to standard care compared to standard care alone for the treatment of persistent auditory hallucinations in people with schizophrenia in Australia.

## Methods

### Analytic approach

The current analysis was undertaken within a broader research program aiming to use economic evidence to inform mental health service delivery within Australia. The program adapted the technical methods of the assessing cost-effectiveness (ACE) Prevention study to improve comparability across cost-effectiveness evaluations [6–8].

A cost-utility framework was used whereby outcomes were expressed as disability-adjusted life years (DALYs) averted. DALYs are a composite measure of premature death (years of life lost, YLL) and years of life lived with a disability (YLD) [9] used in economic evaluations as a measure of population health loss, to assess the incremental benefit of one intervention over another, and to optimize the mix of services provided [10].

This economic evaluation was conducted using an Australian health-system perspective, with a focus on government (Commonwealth and states/territories) as a third-party payer. The evaluation excluded costs to the private sector, nongovernment organizations, out of pocket costs to patients and carers, as rTMS is not currently part of routine clinical practice, and limited data were available to conduct a full health-sector perspective analysis.

While there is no explicit threshold adopted in Australia for decision making, a cost-effectiveness threshold of $50,000/ DALY averted is adopted in this evaluation [6].

### Population

The target population for receiving rTMS in clinical practice is Australian adults aged 25–65 years with schizophrenia treated in a hospital inpatient or community setting who continue to experience symptoms of disease despite treatment with antipsychotic medicines at an optimized dose. rTMS has been evaluated as add-on treatment to standard care comprised of one or more antipsychotic medicines and a range of psychosocial interventions, therefore, standard care is considered the appropriate comparator.

Table 1 summarizes the input parameters and distributions for uncertainty assumptions. The eligible patient population entering the economic model was estimated by taking the whole Australian population (by age and gender) in 2019 from the Australian Bureau of Statistics [11] and applying the prevalence of schizophrenia in Australia from the global burden of disease (GBD) [12]. Of these patients, an estimated 37.5% will experience hallucinations in a 12-month period [13], and 25% of those patients were estimated to have hallucinations refractory to medications [4].

**Table 1.** Input parameters and uncertainty ranges.

Parameter	Value and uncertainty range	Distribution	Source
Australian population	25–65 year-olds	Fixed	[11]
All-cause mortality^a^	Age and gender specific	Fixed	[32]
Schizophrenia specific mortality	1.56	Fixed	[33]
Prevalence of schizophrenia^b^	Age and gender specific	Fixed	[12]
3-month probability of hallucinations	11.1% (7.7–14.9%)	Pert	[13]
Proportion treatment resistant	25% (5–45%)	Pert	[4]
3-month probability of relapse with standard care	12.3% (10.2–14.7%)	Beta	[25]
3-month probability of relapse with repetitive transcranial magnetic stimulation	6.7% (4.8–9.4%)	Beta	[5]
Proportion in acute state admitted to hospital	50% (40–60%)	Pert	[18]
Proportion in acute state receiving intensive case management	50% (40–60%)	Pert	[18]
Proportion in acute state receiving routine case management	50% (40–60%)	Pert	[18]
Disability weight schizophrenia acute state^c^	0.778 (0.606–0.9)	Beta	[23]
Disability weight schizophrenia residual state^c^	0.588 (0.411–0.754)	Beta	[23]

a Refer to Table S1 (Supplementary Materials) for age and sex specific mortality rates.

b Refer to Table S2 (Supplementary Materials) for age and sex specific schizophrenia prevalence.

c Disability weights quantify societal preferences for different health states. They range from 0 representing no disability to 1 representing death. This is an inverse scale to utility weights used to calculate Quality Adjusted Life Years (QALYs).

### Model structure

**Figure 1.**
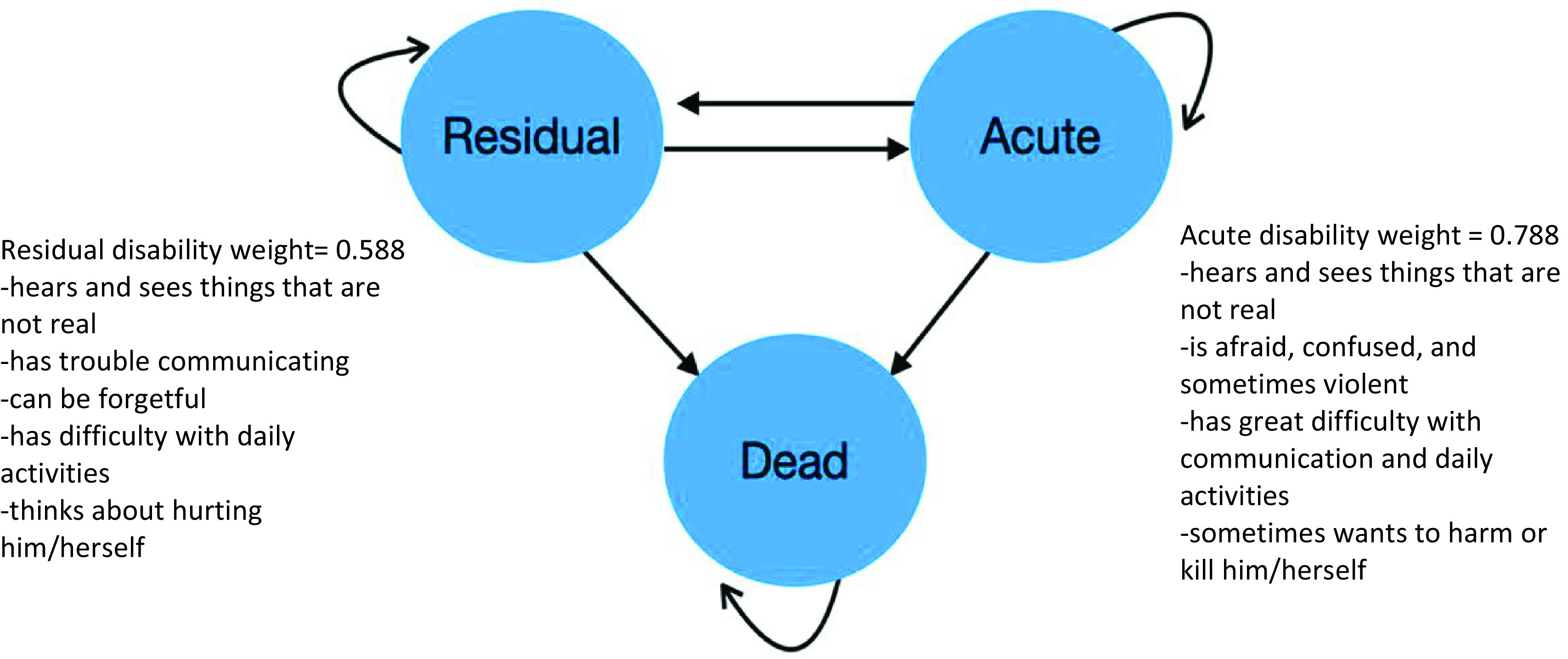
State transition diagram used to estimate the health outcomes associated with rTMS for people with schizophrenia.

A Markov cohort model was developed in Excel to undertake this cost-utility analysis (CUA). Markov cohort models are the most commonly used economic models in schizophrenia [14] and synthesize a range of clinical and epidemiological inputs based on the probability of a population of people moving between health states [15]. The health states in the economic model were aligned with the 2019 GBD health state descriptors, with the corresponding disability weights applied to patients in each modeled health state to calculate total DALYs averted with treatment compared to standard care (Figure 1). These health states are a simplistic reflection of the clinical disease course, whereby patients have periods of relative stability with intermittent relapses of acute episodes [15]. As treatment with rTMS is most appropriate for patients who have continuing hallucinations but otherwise good disease insight, all eligible patients enter the model in the residual health state. In each 3-monthly cycle, patients can remain in the residual health state, transition to the acute health state, or die. Similarly, patients in the acute health state can recover and return to the residual health state, remain in the acute health state, or die. Patients in the acute health state stop receiving rTMS treatment since it is assumed that these patients would lack the required disease insight and stability in mental state to be able to tolerate and adhere to the treatment course. The 3-month Markov cycles were based on the longest follow-up data on rTMS in schizophrenia [5]. The base case analysis covers a 12-month time horizon with no discounting applied. The sensitivity analyses with extended time horizons beyond 12 months use an annual discount rate of 3% for costs and outcomes.

### Intervention description and effectiveness

A range of treatment schedules can be used for rTMS including treatment once or twice daily for 4–30 sessions. In clinical practice, it is unlikely that patients will only receive an intensive treatment course followed by no treatment, particularly if the patient is responding. Instead, it is anticipated that most patients commence a tapering treatment course, for example, once weekly sessions for the first 6 months, followed by once fortnightly sessions. We therefore used a regimen of once daily rTMS for 10 days since this was the most commonly reported regimen from randomized controlled trials of rTMS [5]. This was followed by a tapering treatment course in the base case of the current analysis (Table 2). In the absence of clinical practice guidelines on longer term rTMS treatment in schizophrenia, the tapering regimen was based on expert opinion from a psychiatrist experienced in the use of brain stimulation in the treatment of psychiatric disorders including schizophrenia.

**Table 2.** Repetitive transcranial magnetic stimulation treatment schedule and costs applied in the model.

Treatment week	Treatment protocol	Cost
Week 1	Treatment prescription by psychiatrist Treatment administration once daily for 5 days	$186.40
		$160 × 5 = $800
Week 2	Treatment administration once daily for 5 days	$160 × 5 = $800
Week 3–13	Treatment administration once weekly	$160 × 11 = $1,760
*Total cost–Cycle 1*		*$3,546*
Week 14–26	Treatment administration once weekly	$160 × 13
*Total cost–Cycle 2*		*$2,080*
Week 27–39	Treatment administration once fortnightly	$160 × (13/2)
*Total cost–Cycle 3*		*$1,040*
Week 40–52	Treatment administration once fortnightly	$160 × (13/2)
*Total cost–Cycle 4*		*$1,040*
**Total cost over 1 year**		**$7,706**

To determine the effectiveness of rTMS compared to standard care in the treatment of hallucinations in schizophrenia, a recent high quality meta-analysis of rTMS was identified [5]. This meta-analysis comprised studies evaluating rTMS as an add-on to treatment as usual (TAU) and compared to sham stimulation added to TAU. The main outcome measured was the composite hallucination score, and a statistically significant treatment effect for reduction in hallucinations of −0.51 (*p* = 0.0001), favoring rTMS over sham was observed.

### Resource use and costs

The cost of $160 per rTMS treatment was based on an application to the Medical Services Advisory Committee (MSAC) approved in 2019 to list rTMS on the Medicare Benefits Schedule (MBS) for the treatment of major depression [16]. This is the same as the current private cost per rTMS treatment session [17].

The resource use for standard care of people with schizophrenia was identified by model health state and measured from descriptions of optimal treatment recommendations for schizophrenia from Andrews et al. [18] based on an expert panel’s opinions (Table 3). The panel estimated the proportions likely to receive specific treatments (i.e., hospital admission) and the number of services by remission status. Length of inpatient stay was derived from the 2010 Survey of High Impact Psychosis [19]. Unit costs for medicines were sourced from the Pharmaceutical Benefits Scheme (PBS) [20], unit costs for GP consultations, psychiatrist consultations, CBT and family education were sourced from the MBS [21], and unit costs for community mental health and inpatient admissions were sourced from the second Australian National Survey of Psychosis [2]. All unit costs were inflated to 2021 prices using the health price index [22]. Health state costs were calculated as a weighted average based on the proportion of the population using each service (Table 3) and applied to both rTMS and standard care arms of the model.

**Table 3.** Resource use and cost of standard care by model health state.

Resource	Measurement	Unit cost	Source and assumptions
Residual health state			
Medication	90% take medication; of these: − 67.5% oral risperidone − 13.5% depot risperidone − 9% clozapine	4 mg risperidone, 60: $41.78 37.5 mg risperidone injection, 2: $352.56 100 mg clozapine, 100: $259.86	Percent and type of medication use [18]; Average medication doses [3]; Unit costs [20]
Community mental health (CMH) consults	50% seen through CMH/GP model, receiving 13 CMH sessions per year; 10% treated through private psychiatry receive nine CMH consults per year	$316.26 per CMH consult	Percent and number of CMH use [18]; Unit cost [2] inflated to 2021 $A [22]
GP consultations	50% receive nine consultations per year; 10% treated through private psychiatry receive six GP consults per year	$39.10	Percent and number of GP services [18]; Unit cost for MBS Item 23 (20-min consultation) [21]
Private psychiatrist	10% visit a private psychiatrist 12 times per year	$140.55	Percent and number of psychiatrist services [18]; Unit cost for MBS Item 304 (30–45 min consultation) [21]
Cognitive behavior therapy (CBT)	50% seen through CMH/GP model, of these 40% receive 10 sessions per year of CBT	$145.06	Percent and number of sessions [18]; Unit cost is weighted average price based on two-third people billed under MBS item 80,110 (psychologist, >50 min) and one-third under MBS item 80010 (clinical psychologist, >50 min) [2, 21]
Family education	50% seen through CMH/GP model, of these: 50% receive 13 sessions of family education per year	$122.35	Percent and number of sessions [18]; Unit cost MBS item 170 (Group family therapy) [21]
Acute health state			
Medication	100% take medication; of these: – 20%: depot risperidone – 60%: clozapine – 20%: multiple drug therapy	50 mg risperidone injection, 2: $431.86 200 mg clozapine, 100: $511.94	Percent and type of medication use [18]; Average medication doses [3]; Unit costs [20]; Multiple drug therapy assumed to be risperidone + clozapine
Inpatient admission (public)	50% would have inpatient admission; − 25.4%: 7 days – 22.1%: 21 days – 43%: 45 days	$1,424.73 per day	Percent admitted [18]; Length of stay [13]; Unit cost [2] inflated to 2021 values [22]
Intensive case management	50% receive intensive case management consisting of: – 100 CMH consults per year – Four GP visits per year	As above	Percent and number of sessions [18]; Unit cost [2] inflated to 2021 $A [22]; Unit cost for MBS Item 23 (20-min consultation) [21]
Routine case management	50% receive routine case management consisting of: – 26 CMH consults per year – Four GP visits per year	As above	Percent and number of sessions [18]; Unit cost [2] inflated to 2021 $A [22]; Unit cost for MBS Item 23 (20-min consultation) [21]

### Health outcomes

The 2019 GBD study [23] describes two severity levels for schizophrenia: acute with a disability weight of 0.778 (95%CI 0.606–0.9) and residual with a disability weight of 0.588 (95%CI 0.411–0.754).

While rTMS may impact positive and negative symptoms of schizophrenia, a conservative assumption was made that there would only be a benefit on hallucinations (positive symptoms).

### Transition probabilities

The transition probability of moving from the residual to acute health state varies by whether the cohort receives rTMS or standard care. This required the effect sizes to be converted into relative risk using the Cochrane conversion method [24]. This first required an estimation of the background risk of relapse, which was not included as part of the reporting in Kennedy et al. [5]. Instead, a background risk of relapse of 230 per 1,000 was incorporated into the model from a Cochrane review of maintenance treatment with antipsychotic drugs [25]. This was reflective of the standard care comparator arm. Using the conversion method outlined in the Cochrane handbook resulted in a relative risk of 0.549 (95%CI: 0.39–0.76) for treatment with rTMS. This was then applied to the 3-monthly rate of relapse for the standard care arm and converted to a probability, resulting in a 3-month probability of relapse for the rTMS arm of 6.9%.

### Uncertainty and sensitivity analyses

Probabilistic sensitivity analysis (PSA) evaluated the effect of uncertainty in parameters on cost-effectiveness results. As shown in Table 1, the PSA parameters included were 3-month probability of hallucinations, proportion of the cohort estimated to be treatment resistant, 3-month probability of relapse with standard care, 3-month probability of relapse with rTMS, the proportion of cohort admitted to hospital, proportions receiving intensive case management or routine case management when in an acute state, and disability weights. Ersatz (version 1.35 Sunrise Beach, Australia) was used to run 5,000 iterations and calculate 95% uncertainty intervals (UI) for total costs, DALYs averted and incremental cost-effectiveness ratios (ICERs).

Additional univariate sensitivity analyses determined the effect of variables on cost-effectiveness. Due to the high upfront cost of rTMS sessions, the model is likely to be particularly sensitive to time horizon used and was extended to 5 years. In the extrapolated models, treatment is assumed to continue every fortnight with associated costs accrued, and treatment benefits continuing as observed in the Kennedy et al. [5] meta-analysis.

Inpatient hospital costs contribute the major cost difference between the residual and acute health states. More recent Australian hospital data estimates a 8.5-day average length of hospital stay for schizophrenia with major complexity (AR-DRG U61A) at an average cost of $14,745 in 2021 $A [26]. This cost was used in a univariate sensitivity analysis.

In its consideration of MBS funding for rTMS for use in treatment resistant depression, MSAC noted a lack of information available on the chosen cost of treatment ($160 per administration) [16]. The fee was varied in one-way sensitivity analyses between $100 and $160 in increments of $10.

Additional one-way sensitivity analysis, varying the effect size associated with rTMS treatment, was undertaken to identify a threshold where rTMS would be cost-effective.

## Results

The base-case cost-effectiveness analysis for rTMS over a 12-month time horizon estimated a mean ICER of $87,310 per DALY averted for rTMS as add-on therapy to standard care compared to standard care alone (Table 4). However, these results demonstrate a wide uncertainty interval, from $10,157 to $97,877 per DALY averted.

**Table 4.** Results of the base-case and sensitivity analyses for repetitive transcranial magnetic stimulation compared to standard care.

	Incremental costs mean (95% UI)	DALYs averted mean (95% UI)	ICER mean (95% UI)	Probability CE at $50,000/DALY averted (%)^a^
$160 per session (Base-case)	$31,278 ($693–$77,915)	0.36 (0.07–0.80)	$87,310 ($10,157–$97,877)	28
$150 per session	$26,928 (−$4,355–$70,173)	0.36 (0.07–0.80)	$74,834 (Dominant–$87,805)	35
$140 per session	$22,294 (−$9,384–$65,245)	0.36 (0.07–0.80)	$61,721 (Dominant–$82,754)	44
$130 per session	$17,645 (−$13,805–$58,748)	0.36 (0.07–0.80)	$48,580 (Dominant–$73,209)	52
$120 per session	$12,335 (−$20,439–$51,818)	0.36 (0.07–0.82)	$33,590 (Dominant–$63,319)	61
$110 per session	$8,077 (−$24,356–$46,139)	0.36 (0.07–0.78)	$22,230 (Dominant–$58,812)	68
$100 per session	$3,307 (−$30,815–$39,785)	0.36 (0.07–0.78)	$9,127 (Dominant–$50,699)	74
2-year time horizon	$20,804 (−$48,279–$102,005)	0.69 (0.11–1.56)	$30,300 (Dominant–$65,422)	62
3-year time horizon	$10,194 (−$91,801–$119,584)	0.98 (0.11–2.20)	$10,433 (Dominant–$54,281)	72
4-year time horizon	−$1,727 (−$146,245–$141,516)	1.22 (0.11–2.88)	Dominant (Dominant–$49,163)	77
5-year time horizon	−$12,457 (−$195,350–$167,554)	1.44 (0.07–3.47)	Dominant (Dominant–$48,334)	78
Hospitalization cost reduced	$51,835 ($20,137–$93,644)	0.36 (0.07–0.78)	$143,928 ($258,447–$119,471)	1

a The probability of cost-effectiveness is estimated based on the model results which simultaneously vary the input parameters shown in Table 1.

**Figure 2.**
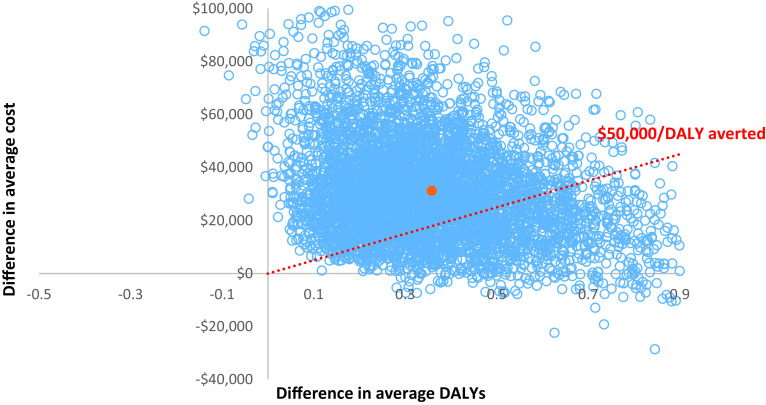
Cost-effectiveness plane for base-case analysis.

The 5,000 iterations of the base-case model results were plotted on a cost-effectiveness plane against a willingness to pay threshold of $50,000/DALY averted (Figure 2). Nearly all iterations (97%) fall in the northeast quadrant where rTMS is associated with additional costs and additional DALYs averted over standard care.

**Figure 3.**
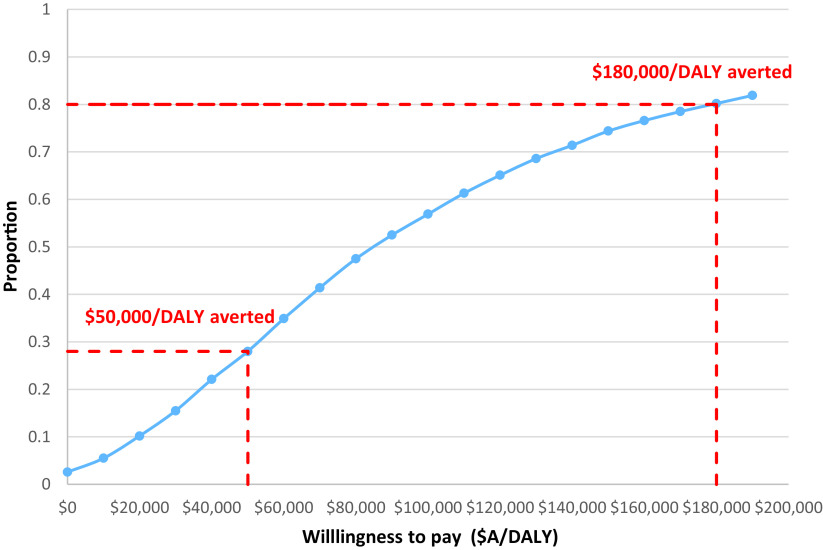
Acceptability curve for the base case analysis.

To determine the probability that rTMS is cost-effective compared to standard of care, and visually represent the level of uncertainty, a cost-effectiveness acceptability curve was developed (Figure 3). At a cost-effectiveness threshold of $50,000/DALY averted, there is a 28% chance that rTMS added to standard therapy is cost-effective compared to standard therapy alone. This probability increases to 80% when the cost-effectiveness threshold is increased to $180,000/DALY averted.

The results of one-way sensitivity analyses are shown in Table 4. Reducing rTMS treatment costs to $100 per session results in the average ICER falling to $9,127/DALY averted (95% UI: Dominant [more effective and less costly]—$50,699/DALY averted). At this cost per session, 74% of the iterations fall below $50,000/DALY averted compared to standard care alone.

When the time horizon was extended to 4 years or more the average ICER becomes dominant (more effective and less costly). Although the uncertainty intervals around the ICER do not cross the $50,000/DALY averted threshold, the probability of adjunctive rTMS being cost-effective relative to standard care ranges from 77 to 78%.

When the cost of inpatient hospital care, which is part of the cost for patients in the acute health state, is reduced, from a base case of $36,714 to $14,745, the ICER increases to $143,928/DALY averted. At the willingness to pay threshold of $50,000/DALY averted, there is a 1% probability of rTMS being considered cost-effective compared to standard care alone in this scenario.

Decreasing the 3-month probability of relapse associated with rTMS treatment from the base case value of 6.7 to 4.6% provides an ICER of $27,811/DALY averted (95% UI Dominant–$51,483) and a 71% probability of rTMS being cost-effective relative to the $50,000/DALY averted willingness to pay threshold.

## Discussion

This is the first cost-effectiveness analysis of rTMS for the treatment of persistent auditory hallucinations in patients with schizophrenia. The base-case results indicate that rTMS is not cost-effective as an add-on therapy to standard care compared to standard care alone for the treatment of adult patients with schizophrenia experiencing treatment-resistant hallucinations at a $50,000 per DALY averted cost-effectiveness threshold.

Adopting a commonly used cost-effectiveness threshold of $50,000 per DALY averted may not be appropriate in the context of this treatment. As the target population for rTMS treatment consists of patients refractory to other treatments who have no other treatment options, and because the disability weight associated with acute schizophrenia is so significant comparative to other diseases, it may be reasonable for decision-makers to adopt a higher ICER threshold. This is consistent with the Rawlsian theory of maximin that can be applied to priority setting whereby the degree of severity of illness is used as a decision-making criteria [27]. A related concept is adopted in Australia whereby the decision context is modified for medicines seeking public subsidy where no alternatives exist for severe progressive medical conditions [28]. While not directly applicable to the population considered in this project, this highlights a potential willingness to accept a higher cost-effectiveness threshold for effective treatments for patients with severe disease who have no alternative treatment options.

Similarly, given treatment is likely to be used as a last-line option in relatively few patients (estimated at approximately 2,500 eligible patients in this cohort model), the total financial impact may justify a higher ICER. A study by Harris et al. [29] analyzed the influence that a range of factors had on decision making for new drug subsidy in Australia, and reported that the higher the total budget cost, the less likely public subsidy would be recommended.

As there are no existing economic evaluations of rTMS for schizophrenia, the results of this project were compared to the modeled cost-effectiveness results of electroconvulsive therapy (ECT) when used in treatment resistant schizophrenia for the purpose of public reimbursement decision making in the UK [30]. ECT was not cost-effective compared to clozapine but was cost-effective compared to haloperidol/chlorpromazine. There was a significant amount of uncertainty regarding these results, however on balance, it was determined that ECT and pharmacological treatments were of equivalent cost-effectiveness and ECT was recommended as a treatment option for rapid improvement of severe symptoms in refractory patients.

The 12-month modeled time horizon for the base-case required extrapolation of treatment duration and effectiveness from clinical trial data, as the longest duration of follow-up in the literature was 3 months [31], and most of the clinical trials included in the meta-analysis were 4 weeks or less. The base-case time horizon can be justified in the context of schizophrenia being a life-long illness and supported by the assumption that patients who respond well to an upfront treatment course of rTMS would likely continue to receive ongoing treatment. A 12-month time horizon would ensure sufficient time to model the impact of relapse from the residual to the acute health state which would not be adequately captured using a shorter time horizon matching the clinical trial duration. Nevertheless, rTMS is a new treatment option in this patient population and longer-term treatment effects including whether the treatment effects would be sustained over the longer term, are as yet unknown so any extrapolation beyond the clinical trial results introduces uncertainty. More information regarding the long-term use of rTMS for this indication will provide greater confidence in the cost-effectiveness estimates.

Inpatient costs are a large contributor to the cost of illness in schizophrenia and are a key driver of the difference in health state costs for this analysis. From a health system perspective, interventions such as rTMS that focus on reducing the time spent in the acute health state avoiding inpatient costs are likely to be an important focus of economic evaluation.

Given the base case assumptions in the current analysis, the 3-month probability of relapse with rTMS treatment would need to decrease from 6.9 to 4.6% for the average ICER and uncertainty intervals to fall below the generally accepted willingness to pay threshold. This would be achieved with an increase in effect size from −0.51 to −0.77. It is unclear if this could be achieved but may be possible in the future as stimulation techniques are researched and refined.

### Limitations

Being a new treatment option for this patient population, there is limited consensus on the correct delivery method (both site of administration and pulse frequency) and the correct treatment regimen. This resulted in clinical trial efficacy results with significant variability contributing to wide uncertainty intervals in cost-effectiveness results. It is appropriate that the most common treatment regimen observed in the meta-analysis was adopted to inform the cost of rTMS; however, the lack of consensus on the optimal treatment course in terms of number and frequency of sessions provides uncertainty in cost estimates.

In the absence of clinical practice guidelines on longer term rTMS treatment in schizophrenia, the tapering treatment course in the base case of the analysis was based solely on expert opinion, a significant limitation of this analysis. Additional trial and observational data may lead to national guidelines on long term treatment courses with rTMS in people with schizophrenia guiding future economic evaluations.

Furthermore, only patients responding to treatment in the initial treatment phase would continue to use rTMS in a tapering course, but as no data was available on nonresponders, this has not been accounted for in the model. Similarly, noncompliance to treatment or voluntary withdrawal was not modeled.

The modeled background risk of relapse between the residual and the acute health states was sourced from a study of all schizophrenia patients on antipsychotic treatment [25] and was likely to underestimate the background rate of relapse for patients not responding adequately to treatment.

The transition probability used in the model for patients in the acute state remaining there in the subsequent cycle is based on length of inpatient stay observed in the Survey of High Impact Psychosis and was considered the best available input; however, no data is available to verify that patients who had a prolonged inpatient stay did so because of an ongoing acute episode. Some patients may have prolonged inpatient stays for reasons other than medical treatment, such as inpatient rehabilitation programs, lack of social support or no fixed address.

An important aim of schizophrenia treatment at all disease stages is the avoidance of relapse into acute psychotic episodes. However, the current model may not adequately capture reductions in symptom intensity and frequency from rTMS that are clinically meaningful outcomes for patients with schizophrenia.

The availability of only two health states reported in the GBD study for calculating DALYs does not reflect the significant heterogeneity in disease presentation, which may limit the clinical relevance of the evaluation.

The lack of memory in the current Markov model means that there is an equal chance of an event occurring regardless of what has occurred in previous cycles [15]. This is unlikely to be reflective of real-world experience in schizophrenia, as duration of time free from relapse and number of previous hospitalizations may predict future relapse and hospitalization. There is also significant heterogeneity in the clinical disease course for schizophrenia patients, making it difficult to represent in an economic model [15].

## Conclusion

Based on the average ICER from our analysis, using a threshold of $50,000/DALY averted, rTMS would not be considered cost-effective over a 1-year period. However, there is a high level of uncertainty in the results based on the current evidence available. Decreasing the administration cost from $160 to $100 per session provided results with uncertainty intervals below the threshold of $50,000/DALY averted.

rTMS is a promising intervention in psychiatry. Since the current case for public reimbursement in Australia for this specific indication demonstrates some uncertainty in the results, there remains value in conducting future research into this treatment option. Updating this economic evaluation with longer-term clinical efficacy data would likely increase the certainty in results.

## Data Availability

Data supporting the findings of this study can be found in Tables 1–3. The data are also available on request from the study authors.

## Supplementary Materials

To view supplementary material for this article, please visit http://doi.org/10.1192/j.eurpsy.2022.13. click here to view supplementary material

## Abbreviations

ACE: assessing cost-effectiveness
AUD: Australian dollars
CBT: cognitive behavioral therapy
CUA: cost-utility analysis
DALYs: disability-adjusted life-years
ECT: electroconvulsive therapy
GBD: global burden of disease
ICER: incremental cost-effectiveness ratio
MBS: Medicare Benefits Schedule
MSAC: Medical Services Advisory Committee
PBS: Pharmaceutical Benefits Scheme
rTMS: repetitive transcranial magnetic stimulation
TAU: treatment as usual
tDCS: transcranial direct current stimulation
UI: uncertainty interval
YLD: years lived with disability
YLL: years of life lost

## References

[1] Charlson FJ, Ferrari AJ, Santomauro DF, Diminic S, Stockings E, Scott JG, et al. Global epidemiology and burden of schizophrenia: findings from the global burden of disease study 2016. Schizophr Bull. 2018;44(6):1195–203. 29762765 10.1093/schbul/sby058 PMC6192504

[2] Neil AL, Carr VJ, Mihalopoulos C, Mackinnon A, Morgan VA. Costs of psychosis in 2010: findings from the second Australian National Survey of psychosis. Aust N Z J Psychiatry. 2014;48(2):169–82. 24097844 10.1177/0004867413500352

[3] Galletly C, Castle D, Dark F, Humberstone V, Jablensky A, Killackey E, et al. Royal Australian and New Zealand college of psychiatrists clinical practice guidelines for the management of schizophrenia and related disorders. Aust N Z J Psychiatry. 2016;50(5):410–72. 27106681 10.1177/0004867416641195

[4] Shergill SS, Murray RM, McGuire PK. Auditory hallucinations: a review of psychological treatments. Schizophr Res. 1998;32(3):137–50. 9720119 10.1016/s0920-9964(98)00052-8

[5] Kennedy NI, Lee WH, Frangou S. Efficacy of non-invasive brain stimulation on the symptom dimensions of schizophrenia: a meta-analysis of randomized controlled trials. Eur Psychiatry. 2018;49:69–77. 29413808 10.1016/j.eurpsy.2017.12.025

[6] Carter R, Vos T, Moodie M, Haby M, Magnus A, Mihalopoulos C. Priority setting in health: origins, description and application of the Australian assessing cost-effectiveness initiative. Expert Rev Pharmacoecon Outcomes Res. 2008;8(6):593–617. 20528370 10.1586/14737167.8.6.593

[7] Mihalopoulos C, Carter R, Pirkis J, Vos T. Priority-setting for mental health services. J Ment Health. 2013;22(2):122–34. 23323752 10.3109/09638237.2012.745189

[8] Whiteford H, Harris M, Diminic S. Mental health service system improvement: translating evidence into policy. Aust N Z J Psychiatry. 2013;47(8):703–6. 23814069 10.1177/0004867413494867

[9] Voigt K, King NB. Disability weights in the global burden of disease 2010 study: two steps forward, one step back? Bull World Health Organ. 2014;92(3):226–8. 24700983 10.2471/BLT.13.126227 PMC3949595

[10] Tan-Torres Edejer T, Baltussen RMPM, Adam T, Hutubessy RCW, Acharya A, Evans DB, et al. Making choices in health: WHO guide to cost-effectiveness analysis. Geneva: World Health Organization; 2003.

[11] Australian Bureau of Statistics. 3101.0 - Australian demographic statistics, December 2019, https://www.abs.gov.au/statistics/people/population/national-state-and-territory-population/dec-2019; 2019 [accessed 28 January 2022].

[12] Global Burden of Disease Collaborative Network. Global burden of disease study 2019 (GBD 2019) results. Seattle, WA: Institute for Health Metrics and Evaluation (IHME), http://ghdx.healthdata.org/gbd-results-tool; 2020 [accessed 18 January 2022].

[13] Morgan VA, Waterreus A, Jablensky A, Mackinnon A, McGrath JJ, Carr V, et al. People living with psychotic illness in 2010: the second Australian national survey of psychosis. Aust N Z J Psychiatry. 2012;46(8):735–52. 22696547 10.1177/0004867412449877

[14] Németh B, Fasseeh A, Molnár A, Bitter I, Horváth M, Kóczián K, et al. A systematic review of health economic models and utility estimation methods in schizophrenia. Expert Rev Pharmacoecon Outcomes Res. 2018;18(3):267–75. 29347854 10.1080/14737167.2018.1430571

[15] Heeg BM, Damen J, Buskens E, Caleo S, de Charro F, van Hout BA. Modelling approaches: the case of schizophrenia. PharmacoEconomics. 2008;26(8):633–48. 18620458 10.2165/00019053-200826080-00002

[16] Medical Services Advisory Committee. Application no. 1196.3 – repetitive transcranial magnetic stimulation (rTMS) for the treatment of depression-resubmission, http://www.msac.gov.au/internet/msac/publishing.nsf/Content/4AB8D2435C6FDCE8CA2584A100816BBA/$File/1196.3%20-%20rTMS-Final%20PSD.pdf; 2019 [accessed 22 August 2021].

[17] Black Dog Institute. Repetitive transcranial magnetic stimulation (rTMS), https://www.blackdoginstitute.org.au/wp-content/uploads/2020/04/tmsfactsheet.pdf?sfvrsn=14; 2016 [accessed 22 August 2021].

[18] Andrews G, The Tolkien II Team. Tolkien II: a needs-based, costed, stepped-care model for mental health services. Sydney, NSW: World Health Organisation Collaborating Cente for Classification in Mental Health; 2007.

[19] Morgan VA, Waterreus A, Jablensky A, Mackinnon A, McGrath JJ, Carr V, et al. People living with psychotic illness, https://www1.health.gov.au/internet/publications/publishing.nsf/Content/mental-pubs-p-psych10-toc; 2011 [accessed 22 August 2021].

[20] Australian Government Department of Health. Schedule of pharmaceutical benefits December 2013. Canberra: Department of Health; 2012.

[21] Australian Government Department of Health. Medicare benefits schedule book operating from 01 July 2013. Canberra: Department of Health; 2013.

[22] Australian Institute of Health and Welfare. Health expenditure Australia 2014–15. Health and welfare expenditure series no.57. Cat. No. HWE 67. Canberra, Australia; 2016.

[23] Global Burden of Disease Collaborative Network. Global Burden of Disease Study 2019 (GBD 2019) disability weights. Seattle, WA: Institute for Health Metrics and Evaluation (IHME), http://ghdx.healthdata.org/record/ihme-data/gbd-2019-disability-weights; 2020 [accessed 12 January 2022].

[24] Green S, Higgins JPT. Cochrane handbook for systematic reviews of interventions. 9.4.6 Combining dichotomous and continuous outcomes. Version 5.1.0. The Cochrane Collaboration. https://training.cochrane.org/handbook/archive/v5.1/; 2011 [accessed 28 January 2022].

[25] Ceraso A, Lin JJ, Schneider-Thoma J, Siafis S, Tardy M, Komossa K, et al. Maintenance treatment with antipsychotic drugs for schizophrenia. Cochrane Database Syst Rev. 2020;8:CD008016. 32840872 10.1002/14651858.CD008016.pub3 PMC9702459

[26] Independent Hospital Pricing Authority. National hospital cost data collection report, public sector, round 23 (financial year 2018–19), https://www.ihpa.gov.au/publications/national-hospital-cost-data-collection-report-public-sector-round-23-financial-year; 2021 [accessed 28 February 2022].

[27] Olsen JA. Theories of justice and their implications for priority setting in health care. J Health Econ. 1997;16(6):625–39. 10176776 10.1016/s0167-6296(97)00010-6

[28] Pharmaceutical Benefits Advisory Committee. PBAC guidelines section 5.4 - Basis for any claim for the ‘rule of rescue’, https://pbac.pbs.gov.au/section-5/5-4-basis-for-any-claim-for-the-rule-of-rescue.html; 2016 [accessed 22 August 2021].

[29] Harris AH, Hill SR, Chin G, Li JJ, Walkom E. The role of value for money in public insurance coverage decisions for drugs in Australia: a retrospective analysis 1994–2004. Med Decis Mak. 2008;28(5):713–22. 10.1177/0272989X08315247 18378939

[30] National Institute for Health and Care Excellence. Guidance on the use of electroconvulsive therapy: technology appraisal guidance [TA59], https://www.nice.org.uk/guidance/ta59; 2003 [accessed 22 August 2021].

[31] Bais L, Vercammen A, Stewart R, van Es F, Visser B, Aleman A, et al. Short and long term effects of left and bilateral repetitive transcranial magnetic stimulation in schizophrenia patients with auditory verbal hallucinations: a randomized controlled trial. PLoS One. 2014;9(10):e108828. 25329799 10.1371/journal.pone.0108828 PMC4203691

[32] Australian Bureau of Statistics. Deaths, year of occurrence, age at death, age-specific death rates, sex, states, territories and Australia, https://stat.data.abs.gov.au/Index.aspx?DataSetCode=DEATHS_AGESPECIFIC_OCCURENCEYEAR; 2019 [accessed 22 August 2021].

[33] Magnus A, Carr V, Mihalopoulos C, Carter R, Vos T. Assessing cost-effectiveness of drug interventions for schizophrenia. Aust N Z J Psychiatry. 2005;39(1–2):44–54. 15660705 10.1080/j.1440-1614.2005.01509.x

