# Supplementary material for: Cost-utility analysis of repetitive transcranial magnetic stimulation as add-on therapy to standard care for the treatment of hallucinations in schizophrenia
Source: Eur Psychiatry. 2022 Mar 30;65(1):e22. doi: 10.1192/j.eurpsy.2022.13 (PMC9058442; doi:10.1192/j.eurpsy.2022.13)
Supplement: Supplementary file 1 [file S092493382200013Xsup001.docx]

**Supplementary Materials**

**Cost-utility analysis of rTMS as add-on therapy to standard care for the treatment of hallucinations in schizophrenia**

Hendriks L, Mihalopoulos C, Le LKD, Loo C, Chatterton ML

**Table S1. Age specific, all causes, death rate in Australia by sex for 2019 [1]**

| **Age** | **Males** | **Females** |
| --- | --- | --- |
|  |  |  |
| 0 - 4 | 0.0008 | 0.0007 |
| 5 - 9 | 0.0001 | 0.0001 |
| 10 - 14 | 0.0001 | 0.0001 |
| 15 - 19 | 0.0004 | 0.0002 |
| 20 - 24 | 0.0007 | 0.0003 |
| 25 - 29 | 0.0007 | 0.0003 |
| 30 - 34 | 0.0009 | 0.0004 |
| 35 - 39 | 0.0012 | 0.0006 |
| 40 - 44 | 0.0017 | 0.0009 |
| 45 - 49 | 0.0025 | 0.0014 |
| 50 - 54 | 0.0034 | 0.002 |
| 55 - 59 | 0.0052 | 0.0031 |
| 60 - 64 | 0.0081 | 0.0046 |
| 65 - 69 | 0.0117 | 0.0069 |
| 70 - 74 | 0.0182 | 0.0118 |
| 75 - 79 | 0.0314 | 0.0207 |
| 80 - 84 | 0.0576 | 0.0399 |
| 85 - 89 | 0.108 | 0.0812 |
| 90 - 94 | 0.19 | 0.1579 |
| 95 - 99 | 0.2716 | 0.2523 |
| 100 and over | 0.2542 | 0.4265 |

**Table S2. Schizophrenia prevalence by age and sex for the Australian population in 2019 [2]**

|  | **Prevalence - Male** | | | **Prevalence - Female** | | |
| --- | --- | --- | --- | --- | --- | --- |
| **Age (years)** | **Mean** | **Upper** | **Lower** | **Mean** | **Upper** | **Lower** |
| 0-4 years | 0.00E+00 | 0 | 0.00E+00 | 0.00E+00 | 0 | 0 |
| 5-9 years | 0.00E+00 | 0 | 0 | 0.00E+00 | 0 | 0 |
| 10-14 years | 1.15E-04 | 0.000241166 | 4.56E-05 | 7.79E-05 | 0.00015796 | 2.97032E-05 |
| 15-19 years | 1.45E-03 | 0.002018952 | 0.001051453 | 7.70E-04 | 0.001130572 | 0.000511202 |
| 20-24 years | 6.29E-03 | 0.007224327 | 0.0049261 | 2.97E-03 | 0.00361241 | 0.00228938 |
| 25-29 years | 8.19E-03 | 0.009440518 | 0.00694154 | 4.32E-03 | 0.005263813 | 0.003577028 |
| 30-34 years | 8.54E-03 | 0.009708185 | 0.007501623 | 5.14E-03 | 0.006378958 | 0.004390975 |
| 35-39 years | 8.49E-03 | 0.00981698 | 0.00750239 | 5.95E-03 | 0.00716272 | 0.00515956 |
| 40-44 years | 8.16E-03 | 0.009136847 | 0.00742604 | 6.72E-03 | 0.00761597 | 0.006033945 |
| 45-49 years | 7.61E-03 | 0.0085175 | 0.006955935 | 7.06E-03 | 0.007870065 | 0.00633237 |
| 50-54 years | 6.88E-03 | 0.007837865 | 0.006221452 | 6.93E-03 | 0.007723227 | 0.006071493 |
| 55-59 years | 6.01E-03 | 0.00694988 | 0.005336563 | 6.41E-03 | 0.007194768 | 0.005507615 |
| 60-64 years | 5.05E-03 | 0.005922895 | 0.004438522 | 5.59E-03 | 0.006376022 | 0.004757822 |
| 65-69 years | 4.08E-03 | 0.004882345 | 0.003543605 | 4.69E-03 | 0.005458285 | 0.003928848 |
| 70-74 years | 3.16E-03 | 0.00385231 | 0.002700387 | 3.78E-03 | 0.004495952 | 0.003114493 |
| 75-79 years | 2.35E-03 | 0.00290997 | 0.001932057 | 2.93E-03 | 0.003568205 | 0.002409865 |
| 80-84 years | 1.67E-03 | 0.00211234 | 0.00130344 | 2.19E-03 | 0.002720857 | 0.001744918 |
| 85-89 years | 1.16E-03 | 0.001515905 | 0.000853767 | 1.59E-03 | 0.002026278 | 0.00122689 |
| 90-94 years | 7.89E-04 | 0.00107559 | 0.000527981 | 1.13E-03 | 0.00151301 | 0.000830878 |
| 95 plus | 5.30E-04 | 0.000786718 | 3.01E-04 | 7.96E-04 | 0.001109525 | 0.000524966 |

**References**

1. Australian Bureau of Statistics. Deaths, Year of occurrence, Age at death, Age-specific death rates, Sex, States, Territories and Australia, <https://stat.data.abs.gov.au/Index.aspx?DataSetCode=DEATHS_AGESPECIFIC_OCCURENCEYEAR>;2019 [accessed 22 August 2021].
2. Global Burden of Disease Collaborative Network. Global Burden of Disease Study 2019 (GBD 2019) Results. Seattle, United States: Institute for Health Metrics and Evaluation (IHME), 2020. Available from http://ghdx.healthdata.org/gbd-results-tool. [accessed 18 January 2022].
